# The stigma of mental health problems and other barriers to care in the UK Armed Forces

**DOI:** 10.1186/1472-6963-11-31

**Published:** 2011-02-10

**Authors:** Amy C Iversen, Lauren van Staden, Jamie Hacker Hughes, Neil Greenberg, Matthew Hotopf, Roberto J Rona, Graham Thornicroft, Simon Wessely, Nicola T Fear

**Affiliations:** 1 King's Centre for Military Health Research, King's College London, Weston Education Centre, Cutcombe Road, London, SE5 9RJ, UK; 2 Academic Centre for Defence Mental Health, King's College London, Weston Education Centre, Cutcombe Road, London, SE5 9RJ, UK; 3 Health Service and Population Research Department, King's College London, Institute of Psychiatry, De Crespigny Park, London, SE5 8AF, UK

## Abstract

**Background:**

As with the general population, a proportion of military personnel with mental health problems do not seek help. As the military is a profession at high risk of occupational psychiatric injury, understanding barriers to help-seeking is a priority.

**Method:**

Participants were drawn from a large UK military health study. Participants undertook a telephone interview including the Patient Health Questionnaire (PHQ); a short measure of PTSD (Primary Care PTSD, PC-PTSD); a series of questions about service utilisation; and barriers to care. The response rate was 76% (821 participants).

**Results:**

The most common barriers to care reported are those relating to the anticipated public stigma associated with consulting for a mental health problem. In addition, participants reported barriers in the practicalities of consulting such as scheduling an appointment and having time off for treatment. Barriers to care did not appear to be diminished after people leave the Armed Forces. Veterans report additional barriers to care of not knowing where to find help and a concern that their employer would blame them for their problems. Those with mental health problems, such as PTSD, report significantly more barriers to care than those who do not have a diagnosis of a mental disorder.

**Conclusions:**

Despite recent efforts to de-stigmatise mental disorders in the military, anticipated stigma and practical barriers to consulting stand in the way of access to care for some Service personnel. Further interventions to reduce stigma and ensuring that Service personnel have access to high quality confidential assessment and treatment remain priorities for the UK Armed Forces.

## Background

Less than half of those who return from combat with mental health problems in the Armed Forces seek help for their disorder [1-4]. Given that effective treatments are available and that untreated mental health problems have a substantial impact on both individual wellbeing and operational effectiveness of the fighting force [5], this is a cause for concern. In this paper we explore three barriers to care; the anticipated public stigma of mental health problems, which is a set of ideas, beliefs, and expectations that a person believes that others hold about mental illness [6]; attitudes towards mental health providers and mental health treatments/services; and practical barriers impeding access to services.

Previous studies from both the UK and the US have suggested that stigma and lack of trust/confidence in mental health providers are leading barriers to help-seeking in Service personnel [7-9]. It is well documented that an individual's beliefs about how they will be perceived by others if they present with a mental health problem are powerful determinants of the likelihood of help-seeking should they become unwell [10].

Whilst it is widely reported that barriers to care are an important deterrent, there has not been any research to compare how these perceived barriers differ between regular and reserve forces. This is important since rates of post combat mental health problems in reserve forces are higher consistently across all the UK studies to date [11,12]. There have also been no previous comparisons of barriers to care in those who are still serving versus those who have left the military. This is particularly important in the UK where veterans become the responsibility of an entirely separate healthcare system (the NHS) upon leaving the military, and there is widespread anecdotal concern that veterans, in particular, struggle to access the care they need once they re-enter civilian life [13]. Finally Hoge et al have reported that Service personnel with anxiety/depression are twice as likely to report a series of stigmatising beliefs as those who are well [2]. We know from our previous work that rates of help-seeking for alcohol misuse are particularly low and yet alcohol problems have a high prevalence in military populations [14], and thus we seek to extend Hoge's work to include perceived barrier data by specific common mental disorder diagnoses including alcohol.

Therefore in this paper we systematically examine barriers to care amongst Service personnel in a large cross-sectional sample of the UK military. We aim to:

a) compare barriers to care in regulars, reservists and those who have left the Armed Forces (veterans) and

b) compare barriers to care in those with and without a current mental health diagnosis.

## Method

We conducted a cross-sectional study of 821 participants consisting of a telephone interview which included a structured clinical interview plus details of service utilisation and potential barriers to care. The sample was drawn from a large existing military cohort [11], stratified by deployment history and regular/reserve status. A two-phase survey technique was used and those who were 'cases' based on the General Health Questionnaire 12-item (GHQ-12) at the first stage of the cohort study were over sampled. The method is described in detail elsewhere [12].

### Participants

This study was based on a sample drawn from Phase 1 of the King's Centre for Military Health Research (KCMHR) military health study. Phase 1 was the first phase of a cohort study of UK military personnel in service at the time of the 2003 Iraq War. In total, 4722 regular and reserve personnel who were deployed on the war-fighting phase of the Iraq War and 5550 regular and reserve personnel who were not deployed to Iraq at this time completed a questionnaire about their military and deployment experiences, lifestyle factors and health outcomes. A proportion of the study participants were subsequently deployed on later deployments, whose mission was counter-insurgency rather than war fighting. Full details of the KCMHR military health study and responders can be found in Hotopf et al [11].

The sample frame for the current study was drawn from the pool of individuals who returned completed questionnaires from the phase 1 of the KCMHR military health study and who gave signed consent to be followed up. Those who were likely to be psychiatric cases were identified by selecting those who were cases on the GHQ-12 [15] from the main cohort. In order to have the power to examine service utilisation and barriers to care in those with mental health problems, the study population was weighted towards the unwell; 70% were a random sample of those who scored above the threshold for 'GHQ caseness' (score of greater than 3); and 30% were a randomly selected subgroup of the non-GHQ cases (two-phase survey technique). Within the 'case' and 'non-case' groups we stratified the sample by regular/reserve status (50% regular, 50% reserve), and deployment status (50% deployed on the main fighting period of the Iraq War (TELIC 1), 50% deployed elsewhere or were not deployed) in order to ensure adequate power to make key statistical inferences. In all other respects, group participants were representative of the KCMHR military health study responders with regards to Service branch, age, and rank and in turn the KCMHR military health study was representative of those who deployed to Iraq in 2003. The participation rate was 821 of a potential sample of 1083 participants which represented a response rate of 76%.

### Interview schedule

The telephone interview schedule included the following sections: 1) Deployment experience since 2003; 2) Patient Health Questionnaire (PHQ) [16] with an additional measure for PTSD symptoms (the 4 item Primary Care PTSD or PC-PTSD) [17]; 3) Perceived needs, health service use (based on a modified version of the Client Services Receipt Inventory [18]), receipt of treatment, and barriers to care (detailed below). The only demographic information collected was change in marital status and rank since all other relevant information was collected during phase 1.

We used the PHQ as a diagnostic instrument to measure the prevalence of common mental disorders, which has been validated for telephone use [19]. Standard diagnostic algorithms were used to score the PHQ [16]. For PTSD symptoms, we used a short measure developed for primary care by the National Center for PTSD (PC-PTSD) [17]. The screen has been widely used in US military health studies and is the main measure of PTSD used in the Post Deployment Health Assessment (PDHA) [4] and Post Deployment Reassessment (PDHRA) mandated by the US Department of Defense [20]. To improve the specificity of the measure, we included a lifetime DSM-IV Criterion A1 event screening question taken from the National Comorbidity Study [21]. All interviews were conducted during 2006 and 2007.

### Barriers to care

Barriers to care were assessed by measuring agreement with a series of statements which covered: a) practical barriers to access of care; b) anticipated public stigma; and c) attitudes to mental health care/providers. All participants were asked to rate using a five-level Likert scale 'how much each of the possible concerns might affect your decision to receive mental health counselling or services if you ever had a mental health problem'. These measures were an expanded version of those used by Hoge et al in their cross-sectional study of US combatants [2].

### Analysis

All statistical analyses were undertaken using the statistical software package STATA (version 10.0) [22]. The majority of the data reported are descriptive statistics (percentages with their 95% confidence intervals). To compare barriers to care amongst regulars, reserves, and veterans, and those with and without mental health problems, unadjusted and adjusted odds ratios are presented together with their 95% confidence intervals. Odds ratios were derived using logistic regression analysis and were adjusted for socio-demographic (age, sex, marital status, educational status) and military (rank, Service, deployment status, role) characteristics that were associated with at least one barrier to care statement and the key variable such as serving status or mental health diagnosis. Responses to each barrier to care questions were grouped into 'agree' (strongly agree and agree) and 'disagree' (strongly disagree and disagree). Responses of 'neither agree nor disagree' were excluded from the analysis [23]. The number reporting 'neither agree nor disagree' ranged from 26 (for 'don't have adequate transport') to 188 (for 'my bosses would blame me for the problem'). All analyses took account of the sample weights by using the survey (svy) commands in STATA.

### Ethical Issues

The study received approval from both the King's College Hospital NHS Research Ethics Committee (ref: 05/Q0703/155) and also from the Ministry of Defence (Navy) Personnel Research Ethics Committee (ref: 0522/22).

## Results

### Agreement with statements about access to services, stigma, and attitudes towards mental health care/providers in the overall sample (Table 1)

**Table 1:** Agreement with statements about access to mental health services, stigma of mental illness and perceived barriers to care, number agreeing (n), overall N for each statement* and weighted percentage agreeing (%).

	n	N*	% agreeing
*Access*			
It's difficult to schedule an appointment	251	696	28.8
It would be difficult to get time off work for treatment	206	756	18.7
I don't know where to get help	165	792	16.0
I don't have adequate transport	40	794	4.4
			
*Stigma*			
Members of my unit might have less confidence in me	556	726	73.2
My unit bosses might treat me differently	558	734	71.3
It would harm my career	381	685	47.3
I would be seen as weak by those who are important to me	356	690	41.0
It would be too embarrassing	330	727	37.1
My bosses would blame me for the problem	182	630	15.4
			
*Attitudes to mental health care/providers*			
My visit would not remain confidential	133	708	14.4
I would think less of a team member or colleague if I knew they were receiving mental health counselling	95	721	10.8
I've had bad experiences with mental health professionals	96	647	8.6
I don't trust mental health professionals	64	677	6.0
My bosses discourage the use of mental health services	75	643	5.6
Mental health care doesn't work	27	653	3.6

(Total sample size = 821)

* Excludes those who responded 'neither agree or disagree' and those with missing data

The most commonly endorsed barriers were stigmatising beliefs relating to the attitudes of the workplace, for example 'members of my unit might have less confidence in me' (73.2%) and 'my unit bosses would treat me differently' (71.3%). Other commonly reported workplace-related concerns included 'it would harm my career' (47.3%) and 'I would be seen as weak by those who are important to me' (41.0%). In terms of practical barriers, the most commonly reported was that 'it would be difficult to schedule an appointment' (28.8%). Only 3.6% endorsed the idea that 'mental health care doesn't work', and 8.6% said that they had prior 'bad experiences with mental health professionals'.

### Comparison of barriers to care in regular personnel, reservists, and veterans (Table 2)

**Table 2:** Agreement with statements about access to mental health services, stigma of mental illness and perceived barriers to care, by serving status (regular, reserve or veteran), weighted percentage (%), odds ratio and 95% confidence interval (CI).

	Regulars (n ranges from 240 to 295)		Reserves (n ranges from 222 to 292)			Veterans (n ranges from 164 to 212)	
	
			Odds ratio^a ^ (95% CI)			Odds ratio^c ^ (95% CI)	
					
	%	%	Unadjusted	Adjusted^b^	%	Unadjusted	Adjusted^d^
*Access*							
I don't know where to get help	11.5	18.7	1.76 (0.90-3.46)	3.06 (1.27-7.42)	20.8	1.42 (0.97-2.09)	1.74 (1.14-2.64)
I don't have adequate transport	3.9	2.9	0.74 (0.21-2.63)	1.77 (0.32-9.69)	7.9	1.46 (0.78-2.73)	2.12 (1.09-4.14)
It's difficult to schedule an appointment	20.4	38.7	2.46 (1.40-4.35)	3.63 (1.79-7.34)	28.8	1.26 (0.90-1.75)	1.36 (0.96-1.92)
It would be difficult to get time off work for treatment	14.5	25.2	1.98 (1.08-3.62)	3.37 (1.60-7.09)	16.5	1.08 (0.76-1.54)	1.23 (0.85-1.76)
							
*Stigma*							
It would be too embarrassing	41.7	34.4	0.74 (0.44-1.23)	0.85 (0.43-1.72)	33.5	0.84 (0.62-1.13)	0.87 (0.63-1.21)
It would harm my career	51.5	48.9	0.90 (0.53-1.51)	1.14 (0.55-2.35)	37.6	0.75 (0.55-1.02)	0.72 (0.52-1.02)
Members of my unit might have less confidence in me	78.1	71.1	0.69 (0.38-1.24)	1.12 (0.50-2.50)	67.3	0.76 (0.54-1.07)	0.81 (0.53-1.21)
My unit bosses might treat me differently	72.4	68.8	0.84 (0.48-1.46)	0.77 (0.36-1.65)	73.0	1.01 (0.73-1.42)	1.11 (0.77-1.61)
My bosses would blame me for the problem	12.3	14.8	1.24 (0.60-2.55)	1.64 (0.62-4.33)	21.5	1.39 (0.94-2.07)	1.85 (1.21-2.83)
I would be seen as weak by those who are important to me	36.6	42.2	1.27 (0.75-2.15)	1.68 (0.81-3.48)	46.5	1.23 (0.91-1.66)	1.32 (0.95-1.85)
							
*Attitudes to mental health care/providers*							
Mental health care doesn't work	5.5	0.7	0.13 (0.04-0.41)	0.79 (0.17-3.66)	4.7	0.92 (0.47-1.81)	1.14 (0.63-2.05)
I don't trust mental health professionals	7.6	2.0	0.25 (0.10-0.61)	0.18 (0.04-0.74)	9.9	1.16 (0.68-1.96)	1.22 (0.69-2.21)
My visit would not remain confidential	16.4	13.1	0.77 (0.39-1.52)	1.17 (0.46-2.94)	13.0	0.87 (0.58-1.31)	0.97 (0.64-1.48)
I would think less of a team member or colleague if I knew they were receiving mental health counselling	11.5	8.1	0.68 (0.31-1.52)	0.53 (0.19-1.46)	14.0	1.12 (0.72-1.74)	1.29 (0.81-2.04)
My bosses discourage the use of mental health services	5.3	3.9	0.73 (0.22-2.41)	0.84 (0.11-6.33)	9.3	1.35 (0.81-2.27)	1.33 (0.77-2.29)
I've had bad experiences with mental health professionals	9.1	7.1	0.76 (0.31-1.87)	0.73 (0.15-3.40)	9.9	1.05 (0.64-1.71)	1.22 (0.54-15.9)

(Total sample size = 821).

Those responding 'neither agree or disagree' are excluded (number excluded ranges from 26 to 188)

^a ^For the odds of agreeing among reserves relative to the odds of agreeing among regulars

^b ^Adjusted for sex, rank, educational status, Service, age, deployment status and role

^c ^For the odds of agreeing among veterans relative to the odds of agreeing among regulars

^d ^Adjusted for sex, age and deployment status

In regular personnel, the most commonly endorsed barriers to care are related to stigmatising beliefs. In general, concerns about access to mental health services were less frequent. Attitudes to mental health care/providers again did not appear to be as much of a barrier as the stigma of consulting. The most common attitude which inhibited help-seeking was a concern that 'my visit would not remain confidential'. Few endorsed the statements that 'mental health care does not work' or that 'my boss would discourage the use of mental health services'.

Reservists were more likely than regulars to endorse practical barriers such as 'I don't know where to get help', and report difficulty in 'scheduling an appointment' and in 'getting time off for treatment'. Reservists were less likely to report 'I don't trust mental health professionals' than regular personnel.

Similarly, veterans were more likely to report 'I don't know where to get help' than regular personnel and that 'I don't have adequate transport', and 'my bosses would blame me for my problem'.

### Comparison of barriers to care in those with and without mental health problems (Table 3)

**Table 3:** Agreement with statements about access to mental health services, stigma of mental illness and perceived barriers to care, by whether the respondent had a depressive/anxiety disorder, alcohol abuse or PTSD symptoms, weighted percentage (%), odds ratio and 95% confidence interval (CI).

		Depressive/anxiety disorder (n ranges from 142 to 187)			Alcohol abuse (n ranges from 146 to 191)			PTSD symptoms (n ranges from 84 to 106)		
				
	% agree among those without any diagnosis (n ranges from 357 to 453)	% agree among those with diagnosis	Odds ratio^a ^(95% CI)		% agree among those with diagnosis	Odds ratio^c ^(95% CI)		% agree among those with symptoms	Odds ratio^e ^(95% CI)	
							
			Unadjusted	Adjusted^b^		Unadjusted	Adjusted^d^		Unadjusted	Adjusted^f^
*Access*										
I don't know where to get help	13.0	29.7	2.83 (1.40-5.74)	2.44 (1.04-5.72)	19.9	1.66 (0.85-3.27)	1.02 (0.47-2.19)	33.5	3.38 (1.26-9.04)	3.77 (1.13-12.6)
I don't have adequate transport	2.1	14.1	7.57 (2.25-25.4)	5.45 (1.69-17.6)	11.2	5.80 (1.79-18.8)	1.75 (0.68-4.51)	2.5	1.20 (0.31-4.58)	2.23 (0.44-11.4)
It's difficult to schedule an appointment	26.6	37.1	1.62 (0.85-3.10)	2.03 (1.02-4.05)	37.4	1.65 (0.89-3.03)	2.13 (1.08-4.20)	38.0	1.69 (0.69-4.15)	1.99 (0.69-5.78)
It would be difficult to get time off work for treatment	15.3	38.4	3.45 (1.79-6.66)	3.06 (1.51-6.21)	20.1	1.39 (0.72-2.69)	1.26 (0.58-2.74)	33.3	2.77 (1.17-6.57)	2.74 (1.08-6.92)
										
*Stigma*										
It would be too embarrassing	33.6	54.6	2.37 (1.27-4.43)	1.73 (0.88-3.42)	40.6	1.35 (0.76-2.41)	1.06 (0.57-1.98)	70.5	4.72 (2.15-10.4)	4.21 (1.73-10.2)
It would harm my career	45.3	50.8	1.25 (0.67-2.34)	1.25 (0.63-2.49)	53.1	1.36 (0.75-2.48)	1.49 (0.76-2.91)	57.5	1.63 (0.62-4.27)	2.31 (0.76-7.06)
Members of my unit might have less confidence in me	71.9	68.5	0.85 (0.43-1.67)	0.73 (0.34-1.57)	83.5	1.98 (0.90-4.35)	3.14 (1.36-7.25)	77.9	1.38 (0.47-4.09)	1.47 (0.46-4.68)
My unit bosses might treat me differently	69.7	76.1	1.38 (0.68-2.82)	1.01 (0.45-2.26)	81.5	1.92 (0.91-4.02)	2.01 (0.92-4.40)	76.8	1.44 (0.48-4.29)	1.75 (0.58-5.33)
My bosses would blame me for the problem	11.2	35.3	4.31 (2.05-9.04)	3.41 (1.56-7.42)	24.9	2.62 (1.25-5.52)	2.06 (0.95-4.46)	45.7	6.66 (2.71-16.4)	6.47 (2.63-15.9)
I would be seen as weak by those who are important to me	36.1	57.6	2.41 (1.27-4.55)	2.36 (1.19-4.68)	51.7	1.90 (1.04-3.45)	1.94 (1.00-3.75)	57.3	2.38 (0.95-5.95)	2.34 (0.92-5.96)
										
*Attitudes to mental health care/providers*										
Mental health care doesn't work	2.9	6.3	2.25 (0.43-11.7)	2.18 (0.35-13.5)	5.0	1.79 (0.43-7.40)	2.26 (0.32-16.1)	1.4	0.48 (0.09-2.42)	0.85 (0.14-5.13)
I don't trust mental health professionals	3.7	13.6	4.09 (1.38-12.2)	3.25 (0.81-13.1)	10.8	3.15 (1.06-9.34)	3.30 (0.94-11.6)	11.0	3.24 (1.16-9.05)	2.22 (0.49-9.95)
My visit would not remain confidential	11.4	19.2	1.84 (0.86-3.94)	1.93 (0.86-4.33)	22.2	2.21 (1.05-9.34)	2.41 (1.16-5.03)	21.8	2.15 (0.98-4.74)	2.12 (0.83-5.45)
I would think less of a team member or colleague if I knew they were receiving mental health counselling	7.8	15.9	2.25 (0.89-5.73)	1.81 (0.69-4.75)	22.6	3.47 (1.60-7.51)	2.87 (1.23-6.71)	8.4	1.08 (0.43-2.72)	1.00 (0.26-3.76)
My bosses discourage the use of mental health services	4.6	11.9	2.79 (1.05-7.43)	2.71 (0.99-7.42)	6.0	1.32 (0.49-3.55)	1.09 (0.28-4.22)	19.4	4.95 (1.46-16.7)	2.36 (0.59-9.53)
I've had bad experiences with mental health professionals	7.1	12.7	2.21 (0.89-5.48)	2.27 (0.89-5.83)	19.3	3.61 (1.47-8.84)	4.95 (1.92-12.8)	27.1	5.63 (1.88-16.8)	3.94 (1.18-13.2)

Those responding 'neither agree or disagree' are excluded (number excluded ranges from 26 to 188).

^a ^For the odds of agreeing among those with a depressive/anxiety disorder relative to the odds of agreeing among those without a depressive/anxiety disorder

^b ^Adjusted for age, Service and childhood vulnerability

^c ^For the odds of agreeing among those with alcohol abuse relative to the odds of agreeing among those without alcohol abuse

^d ^Adjusted for age, Service, childhood vulnerability, marital status, medical downgrading status and sex

^e ^For the odds of agreeing among those with PTSD symptoms relative to the odds of agreeing among those without PTSD symptoms

^f ^Adjusted for deployment status, Service, childhood vulnerability and educational status

In terms of access issues, compared to those without any diagnosis, those with a diagnosis of depression were more likely to report that they 'don't know where to get help', 'did not have adequate transport', 'it is difficult to schedule an appointment', and that 'it would be difficult getting time off work for treatment'. Depressed participants were also more likely to report that 'my boss would blame me for the problem', and 'I would be seen as weak by those who are important to me'.

Compared to those without any diagnosis, those with alcohol problems were more likely to endorse 'members of my unit might have less confidence in me', 'my visit would not remain confidential', 'I would think less of a team member if I thought they were receiving mental health counselling' and 'I have had bad experiences with mental health professionals'. They also reported difficulty in scheduling an appointment.

. In comparison to those without any diagnosis, those with PTSD symptoms were more likely to report that they did not know where to get help, and that 'it would be difficult getting time off for treatment'. Those with PTSD were also more likely to endorse 'it would be too embarrassing', and 'my boss would blame me for the problem'. In terms of attitudes to mental illness/providers, those with PTSD were more likely to report 'I have had bad experiences with mental health professionals'.

## Discussion

Four main findings emerge from this study. First, the most common barriers to care reported are those relating to the anticpated public stigma associated with consulting for a mental health problem. Second, barriers to care do not appear to be reduced after people leave the Armed Forces: indeed veterans reported additional barriers to care. Third, reservists believed that they would experience additional difficulties in the practicalities of consulting such as scheduling an appointment and having time off for treatment. Finally, those with mental health problems such as PTSD reported significantly more barriers to care than those who were well.

### Strengths and weaknesses of the study

To our knowledge, this is the first study to date which has examined barriers to care in a representative sample of the UK military.

The strengths of this study are the large sample and high response rate, with no evidence of bias in terms of health between responders and non-responders [12]. The sample is diverse, representing all three services, includes those who have deployed and those who have not, and includes regulars, reservists, and those who have left. The study used a structured clinical interview, and did not rely on questionnaire self-report of symptoms or distress. As data collection took place independent of the military, data quality should have been unaffected by participants' concerns that their problems may have been reported back to the chain of command.

Although our response rate was high, our sample was already based on a 61% response rate [11]. Whilst the indications are that there is no difference in health status between responders and non-responders in the original survey, it is still possible that we missed a small proportion of individuals who made up the most vulnerable, unwell or socially excluded members of population, such as those who were in prison or who were homeless.

### Stigma

Stigma had been reported as an important deterrent for seeking help for mental health problems in the general population [24]. It is likely that such deterrents are amplified in military culture where characteristics of strength, resilience, and self-sufficiency are selected for and prized. Our report that the most significant barrier to seeking mental health care is the anticipated stigma of consulting and lack of trust/confidence in mental health services mirrors what is reported for the US military [2,25], the Canadian military [1,26], and previously for the UK military [3,8]. Taken together these results suggest that despite recent efforts to de-stigmatise mental health problems, a substantial proportion of military personnel still anticipate stigma and believe that any help-seeking behaviour is likely to negatively impact their career and relationships with their seniors.

### Career impact and confidentiality: Valid concerns?

Is there any truth to the assertion that Service personnel who admit to mental disorders will be disadvantaged in their career progression, or somehow viewed more negatively by their peers? There is some limited evidence from the US that commanders view soldiers who have consulted for mental health problems more negatively [27], and this was the perception of some of the participants in this UK study. We also know that mental health problems do have implications for certain occupational roles within the military - for example - whilst suffering from a mental health problem, some personnel will not be allowed to carry weapons, or pilot military aircraft.

Again, whilst confidentiality is a guiding principle, it cannot be absolute as is the case in any branch of occupational medicine. The military chain of command may be informed if a member of their unit is suffering from a health problem, physical or mental, which is deemed to affect their employability, though only essential details which influence their occupational role will be passed on. Thus to some extent, many of the barriers endorsed represent realistic concerns and constitute legitimate barriers to care in the current system but we should point out that similar barriers exist (in the UK) for other professions such as doctors, airline pilots, heavy goods vehicle drivers and so on. Confidentiality may be a particular concern and deterrent to help-seeking in the close-knit environment of the military where medics and their potential patients work alongside each other, live and socialise together.

Access to confidential sources of help have been identified as a key incentive to promote help-seeking in a recent study of soldiers post-deployment [28]. The challenge, therefore, for military health providers is to reduce stigma and maintain confidentiality as much as is practical, whilst providing the military with an occupational health service which not only maintains but maximises operational efficiency and readiness.

### Special barriers for reservists

Reservists report more specific difficulties related to the practicalities of accessing treatment. This is likely to be because those reservists who we interviewed were not mobilised at the time of their interview and had returned to their civilian lives and employment. Our previous work has documented not only that reservists who have deployed are at especially high risk [29] but also that civilian employers of reservists often have little knowledge or understanding of the needs of reservists who have been deployed [30]. In addition, the military peer group which may serve an important function in encouraging help-seeking, and mitigating barriers to care is no longer accessible for reservists on their return home. This would suggest that more recent efforts to provide accessible and bespoke services for reservists are timely and important. The Reservist Mental Health Programme which was implemented during this study is one such initiative although at the present time only small numbers of reservists are accessing this service (personal communication; Norman Jones). Perhaps more important is the education of primary care providers in the likely sequelae of combat and the special challenges of reservists and ensuring that patients who present to primary care are signposted effectively.

### Barriers to care for veterans

Veterans appear to take their barriers to care with them when they return to civilian life, and report additional difficulties of 'not knowing where to seek help', 'not having adequate transport' and the stigma of 'my bosses would blame me for the problem'. There is much interest in the fate of veterans and several anecdotal reports have suggested that veterans experience difficulties in accessing the care that they require [31]. These results confirm that there are barriers to care for veterans which need to be borne in mind when designing and delivering services for those who have left the military. The Community Veteran Pilots currently underway in the UK, which aim to embed a therapist with mental health expertise within a Primary Care Trust who can act as an assessor and advocate for veterans with mental health problems, represent an innovative model of service delivery, an evaluation of which will be published shortly.

### Barriers for those who are unwell and barriers for specific diagnostic groups

In common with Hoge et al [2], we demonstrate that barriers to care are significantly more likely to be reported in those with mental health problems. Individuals who are not experiencing current problems may be less likely to actively consider the consequences of consulting because it is not immediately relevant to them and therefore they report less anticipated stigma [9]. Further, it may also be that people who are currently unwell are more likely to make negative evaluations of services available and are more pessimistic about the prospect and consequences of consulting. Our analysis builds on the existing work of Hoge et al [2] and suggests that different sets of barriers exist for different common mental disorders. Our participants with depression were more likely to endorse practical difficulties with consulting; perhaps because low motivation/energy associated with depression is impairing their ability to initiate consultations. Those with alcohol problems, on the other hand, seem more likely to endorse items associated with shame/stigma. Further understanding barriers to care for specific diagnoses will be important in tailoring intervention strategies towards different groups.

## Conclusions

The implications of this work are three-fold. First, we present clear evidence that the main barrier to seeking help is anticipated public stigma, and therefore interventions aimed at reducing stigma in the military are important and timely. Stigma is learned and culturally specific and an important implication of this is that it may be unlearned [32]. The US military have recently introduced a series of education programmes delivered in a group setting for soldiers and their families, which aim to decrease the stigma associated with seeking help for a mental health problem and encourages soldiers to seek assistance if they have symptoms ([33], and have demonstrated in a randomised controlled trial that for those with high combat exposure there is a reduction in stigmatising beliefs post-intervention (compared with pre- intervention)[34]; a version of the same tool is currently being trialled in the UK. A recent re-examination of the same barriers to care as were reported in Hoge's 2004 study has shown a substantial reduction in the perception of these barriers over the last four years, particularly with respect to the belief that personnel seeking help would be 'seen as weak' (emphasis has been made in education of the chain of command that 'help-seeking is a sign of strength') [28], and this has been more recently confirmed by the Mental Health Advisory Team (VI) report which has showed a reduction in the endorsement of barriers to care statements over time [35]. This is encouraging and suggests that interventions of this kind are capable of beginning to shift stigmatising beliefs and that culture change can be achieved. In the same study, Warner et al reported that the encouragement of friends and family was cited as the most important factor in overcoming barriers to care by Service personnel [28]. Interventions designed to support and educate families are, therefore, a crucial adjuvant to the above, and more research is needed to determine how this can be achieved most effectively.

In addition to this, Greene-Shortridge et al [9], drawing on Corrigan and Penn's model of methods to reduce stigma [36], have suggested a series of measures worthy of consideration. Programmes which aim to promote contact with individuals who have a mental illness could be exploited in a military context by, for example, involving soldiers with PTSD who have been successfully treated in structured discussion and education within the unit, and indeed evaluations of such an initiative are currently underway in Canada where it has proved popular. Green-Shortridge et al [9] also emphasise the importance of encouraging leaders to take an active role in identifying and assisting soldiers to receive mental health support; this may be of particular relevance since fears about leader's view of help-seeking was one of the most highly endorsed stigma statements in this study. Recent studies have shown that leaders in general, contrary to personnel's fears, take a positive attitude towards their staff seeking help [37]. Leaders who make it clear to their subordinates that they endorse the notion that PTSD results from exposure to extreme stressors rather than individual weakness [9], and that help-seeking is acceptable and a sign of maturity, are likely to be powerful agents of change and reduction in stigma [9], and this is confirmed by recent work which has shown that positive leadership and higher unit cohesion reduces stigma and barriers to care, independent of any effect on the prevalence of mental health problems [38]. There is also evidence that individuals who are referred for mental health treatment by the chain of command are much more likely to complete the course of treatment than those who self-refer, indicating that approval is an important catalyst for engagement with treatment [39]. Peer-led schemes which aim to educate leaders in how to identify and signpost vulnerable individuals after a traumatic event such as Trauma Risk Management (TRiM) may well serve this purpose [40]. An important caveat though is that any new interventions must be subjected to rigorous evaluation (for example, a randomised controlled trial) before they are introduced, as if they are ineffective, they may simply serve to medicalise distress without benefit.

As well as general interventions to reduce stigma in the still-serving military, we suggest that specific interventions are required to target those who are most vulnerable. Reservists in particular are experiencing practical barriers to receive of treatment, and therefore educating civilian employers and general practitioners about common mental health problems after deployment whilst emphasising the importance of providing guaranteed time off from work to consult without penalty is important.

In the UK, the care of veterans falls to the National Health Service after they leave the Armed Forces and we suggest again that outreach programmes which aim to educate primary and secondary care about common problems in the veteran population are important so that mental health problems can be identified and treated promptly in this vulnerable group. The stigma associated with consulting is reported to be less if the Service personnel can consult someone who has knowledge and expertise of military matters [28], and hence the service charities have an important role to play in both providing care and in outreach and education of the NHS. Recent initiatives such as the recent MoD/NHS/Combat Stress Community Veteran Pilot Scheme aim to exploit these partnerships further, and are currently being evaluated.

Finally, we report that unwell personnel are the most likely to report barriers to care. This finding has important public health implications for the military as it suggests efforts to target stigmatising beliefs needs to be targeted toward those who are most unwell. Efforts such as outreach and formal education programmes to reduce the stigma of consulting, and clear unambiguous messages from the chain of command that personnel are actively encouraged to seek help targeted at high risk groups (such as those returning from operational duties) are important and timely.

## Competing interests

This study was funded by UK's Ministry of Defence contract number R&T/1/0078. The authors' work was independent of the funding source, and we disclosed the paper to the Ministry of Defence at the point we submitted it for publication. JHH is a Ministry of Defence Civil Servant seconded to the Academic Centre for Defence Mental Health. NG is a full-time active service medical officer and Defence Professor of Mental Health who has been seconded to the Academic Centre for Defence Mental Health and SW is Honorary Civilian Consultant Advisor in Psychiatry to the British Army. All other authors declare that they have no conflicts of interest. All authors, had access to the study data, and NTF and AI take responsibility for the integrity of the data and the accuracy of the data analysis.

## Authors' contributions

AI, NG, SW, LVS, and NTF were responsible for the design of the study. LVS, AI, and JHH undertook data collection for the study. NTF and AI undertook the analysis of the data. AI and NTF prepared the first draft of the manuscript. NG, MH, RR, GT, and SW all had input into developing the manuscript further prior to submission. All authors commented on the manuscript prior to submission and approved the final manuscript.

## Pre-publication history

The pre-publication history for this paper can be accessed here:

http://www.biomedcentral.com/1472-6963/11/31/prepub
